# Histone modifications and chromatin dynamics: a focus on filamentous fungi

**DOI:** 10.1111/j.1574-6976.2007.00100.x

**Published:** 2008-01-25

**Authors:** Gerald Brosch, Peter Loidl, Stefan Graessle

**Affiliations:** Division of Molecular Biology, Biocenter, Innsbruck Medical UniversityInnsbruck, Austria

**Keywords:** chromatin, epigenetic regulation, histone acetylation, protein methylation, filamentous fungi

## Abstract

The readout of the genetic information of eukaryotic organisms is significantly regulated by modifications of DNA and chromatin proteins. Chromatin alterations induce genome-wide and local changes in gene expression and affect a variety of processes in response to internal and external signals during growth, differentiation, development, in metabolic processes, diseases, and abiotic and biotic stresses. This review aims at summarizing the roles of histone H1 and the acetylation and methylation of histones in filamentous fungi and links this knowledge to the huge body of data from other systems. Filamentous fungi show a wide range of morphologies and have developed a complex network of genes that enables them to use a great variety of substrates. This fact, together with the possibility of simple and quick genetic manipulation, highlights these organisms as model systems for the investigation of gene regulation. However, little is still known about regulation at the chromatin level in filamentous fungi. Understanding the role of chromatin in transcriptional regulation would be of utmost importance with respect to the impact of filamentous fungi in human diseases and agriculture. The synthesis of compounds (antibiotics, immunosuppressants, toxins, and compounds with adverse effects) is also likely to be regulated at the chromatin level.

## Introduction

Genomic DNA in the eukaryotic cell nucleus only a few microns in diameter has to be compacted by a factor of *c*.10 000 by the conserved, basic histone proteins to form a highly organized structure termed chromatin. Chromatin is the final result of various processes and phenomena and consists of several levels of organization, ranging from the molecular properties of nucleosomes to the spatial arrangement of chromatin and chromosomes within the nuclear space. Whereas there has been improved knowledge of nucleosome and chromosome structure during the last few years, one is still at the very beginning of understanding how chromatin is functionally connected to the different components of a large-scale nuclear skeleton.

Owing to the organization of DNA in nucleosomes and the resulting compaction, chromatin has long been assessed as an inert structure incompatible with dynamic processes, such as DNA replication, recombination, or transcription. In general, the term ‘structure’ implicates continuous stability; however, chromatin at all compaction levels is not at all static; especially at the level of nucleosomal arrays, chromatin is recognized as highly dynamic and essential for the regulation of all processes taking place at the underlying DNA (Vaquero *et al.*, 2003). The three-dimensional structure of the nucleosome even provides a complex platform for modulating factors to bind through protein–histone interactions, thereby considerably expanding the cell's regulatory repertoire of proteins that bind DNA by sequence specificity only.

There are several mechanisms by which chromatin dynamics is introduced. (1) Chromatin can undergo ATP-dependent remodeling, a process that may also lead to the exchange of histone primary structure variants (Lusser & Kadonaga, 2003; Eberharter & Becker, 2004). (2) Histones are subject to posttranslational modifications with structural and functional consequences (Turner, 2007). (3) DNA itself can be modified by methylation (Dobosy & Selker, 2001). For the modification of DNA by cytosine methylation, it is known that it can be stably inherited to progeny cells. DNA methylation, chromatin remodeling, and chromatin modifications do not act independently from each other but are strongly interrelated *in vivo*. A recent genome-wide analysis of chromatin modifiers demonstrated their essential importance for transcriptional regulation in yeast (Steinfeld *et al.*, 2007).

Filamentous fungi are a heterogeneous group of organisms with the common feature of the production of a huge variety of metabolites; their effects can be beneficial, as in the case of antibiotic compounds (e.g. penicillins), or toxic, as in the case of mycotoxins (e.g. aflatoxin). Given that fungi play an outstanding role in medicine, pharmaceutical production, biotechnology, waste management, and agriculture, it is important to better understand the role of chromatin for transcriptional control mechanisms in these lower eukaryotes.

This review will focus on histone modifications (acetylation, methylation) in filamentous fungi, but also highlight some evidences linking chromatin modifications and signal transduction in general. It will pinpoint differences in chromatin-modifying enzymes between fungi and other eukaryots, because these differences may be exploited in optimizing secondary metabolite (SM) production or developing pharmaceutical compounds for the treatment of fungal infections. It is important to keep in mind that besides universal common features of chromatin modifications in all eukaryotic organisms, distinct differences do exist between phylogenetic kingdoms, as was exemplified for the plant kingdom (Loidl, 2004).

## Chromatin, nucleosomal histones, and their posttranslational modifications

During the last decade, the effects of chromatin structure on the readout of the genetic information of DNA have emerged as a fundamental regulatory level in cellular metabolism. Changes in chromatin structure may lead to short- or long-term alterations of the transcriptional activity of genes, thereby being crucial for the functional properties of cells or tissues. Chromatin modifications are governed and modulated by intrinsic cellular programs but also by extrinsic and environmental factors. They play a crucial role in the maintenance of cellular integrity, differentiation, development and hence disease (Jones & Baylin, 2002; Robertson, 2005), metabolic production (Bok *et al.*, 2006), and environmental adaptation (Grimaldi *et al.*, 2006).

The basic subunit of eukaryotic chromatin is the nucleosome core particle, which consists of 147 base pairs of superhelical DNA wrapped around a core histone octamer in 1.75 turns. An H3/H4-tetramer represents the core element that is flanked by two H2A/H2B dimers (Luger, 2003). Nucleosomes form an 11 nm fiber (‘beads on a string’), which may be further condensed into a 30 nm fiber; this compaction is at least partially augmented by incorporation of the linker histone H1. The highest level of compaction is the metaphase chromosome although the mechanisms leading to this ultimate compaction are poorly understood.

The core histones (H3, H4, H2A, H2B) are highly conserved, basic proteins that consist of a globular domain and a flexible N-terminal tail that does not contribute to the intrinsic structure of the nucleosome (Luger, 2003). H2A and H2B contain another unstructured tail domain at the C-terminus. A large number of amino acids are subject to a variety of posttranslational modifications, most of which are located in the flexible N-terminal tails of the histones. These modifications include acetylation of lysines (e.g. Loidl, 1994; Sterner & Berger, 2000), phosphorylation of serines and threonines (e.g. Nowak & Corces, 2004), methylation of lysines and arginines (e.g. Zhang & Reinberg, 2001; Lachner & Jenuwein, 2002), ubiquitination of lysines (e.g. Davie & Murphy, 1990), ADP-ribosylation of arginines and lysines (Adamietz & Rudolph, 1984), SUMOylation of lysines (Nathan *et al.*, 2003), as well as glycosylation, carbonylation, and biotinylation of different residues (Liebich *et al.*, 1993; Hymes *et al.*, 1995; Wondrak *et al.*, 2000). Among the modifications, acetylation and methylation have an outstanding position, in that they are by far the most extensively studied and are both reversible.

## Linker histone H1

In contrast to core histones, which are highly conserved in eukaryotes, the linker histone H1 exhibits a lower degree of evolutionary conservation. This microheterogeneity was interpreted in the sense that these proteins may be less important or even dispensable for the organization of chromatin. Several studies addressed the functional significance of H1 (e.g. Prymakowska-Bosak *et al.*, 1999; Fan *et al.*, 2005; Wierzbicki & Jerzmanowski, 2005; Jedrusik & Schulze, 2007). However, in higher eukaryotes, the existence of multiple H1 variants complicates the investigation of global H1 functions. In contrast, unicellular organisms like the protozoan *Tetrahymena thermophila* or *Saccharomyces cerevisiae* possess only a single histone H1 form; *Schizosaccharomyces pombe* even lacks H1 completely. However, in these organisms, the amino acid sequence of H1 proteins differs considerably from that of metazoan linker histones (Wu *et al.*, 1986; Shen *et al.*, 1995; Escher & Schaffner, 1997); therefore, a general conclusion of the effects of H1 deletion is questionable with respect to multicellular, higher eukaryotes.

Filamentous fungi are characterized by a single H1 protein with the tripartite structural organization typical for linker histones of multicellular eukaryotes: a winged helix motif flanked by a less structured N-terminal and a basic lysine-rich C-terminal domain (Kasinsky *et al.*, 2001). These facts make filamentous fungi valuable for studying the effects of H1 depletion on chromatin assembly, gene expression and growth. Physiologically interesting phenotypes associated with a completely silenced H1 gene were reported for the ascomycete *Ascobolus immersus*. Using the methylation-induced premeiotically (MIP) technique, the H1 gene from *Ascobolus immersus* was totally silenced, leading to three distinct phenotypic changes: (1) an increase in the overall extent of global DNA hypermethylation, (2) enhanced accessibility of chromatin to micrococcal nuclease, and (3) a significantly reduced life span (Barra *et al.*, 2000).

These results were consistent with an essential role of H1 in global chromatin function; however, they were in contrast to data obtained in the ascomycete *Aspergillus nidulans*, where deletion of the H1 gene *hhoA* did not result in an obvious phenotype (Ramon *et al.*, 2000).

To further clarify the role of H1 in filamentous fungi, hH1 was silenced by the use of the repeat-induced point mutation (RIP) technique (Selker & Stevens, 1985) in *Neurospora crassa*. In contrast to H1 mutants of *Ascobolus immersus*, the absence of H1 in *Neurospora* did not affect global DNA methylation, but caused subtle changes in sensitivity to micrococcal nuclease. Moreover, *hH1* mutants exhibited a slow-growth phenotype dependent on the carbon source used (Folco *et al.*, 2003). It was therefore assumed that H1 might be involved in the expression of different sets of genes in a species-specific manner, in this particular case, of genes of the carbon metabolism in *Neurospora*. Indeed, a pyruvate decarboxylase gene was found to be regulated by histone H1, irrespective of its location along the fungal genome (Folco *et al.*, 2003).

Taken together, these and other data revealed rather different phenotypes of H1-depleted fungal mutant strains and also investigations of other model organisms indicated manyfold functions of H1. However, in multicellular eukaryotes, it becomes more and more evident that, besides a role in a higher order chromatin structure, linker histones are related to gene-specific DNA methylation, which in turn is responsible for the regulation of different DNA regions (e.g. Barra *et al.*, 2000; Fan *et al.*, 2005; Wierzbicki & Jerzmanowski, 2005). Moreover, both lack of H1 and DNA methylation, may affect posttranslational histone modifications (Barra *et al.*, 2005), which in turn may modulate these epigenetic effects.

## Histone code

Acetylation and methylation of histones were first reported almost half a century ago (Allfrey *et al.*, 1964). Subsequently, research in this field was focused on the reversible acetylation reaction and it was assumed for more than two decades that acetylation primarily acts in transcriptional activation through the neutralization of the positive charge of the ɛ-amino group of lysine. Because a total of 26 potentially acetylated lysine residues are present in a nucleosome, this overall charge neutralization might weaken the interaction of the core histone octamer with the negatively charged DNA. However, experimental evidence called this hypothesis into question, in particular, work in the acellular slime mold *Physarum*; based on cell cycle-dependent effects of histone acetylation in this mold, it was proposed that acetylation of lysine residues rather changes the functional properties of the nucleosome by altering the interactions of regulatory proteins with histones than the structure of the nucleosome itself (Loidl, 1988, 1994). Taking into account the various modifications that can occur simultaneously on a nucleosome, in particular at the N-terminal histone tails, it is conceivable that these modifications create a specific pattern that serves as a receptor-like docking station for regulatory factors that recognize and somehow interpret the modification signals. Accordingly, the term ‘histone code’ has been introduced as a counterpart to the ‘genetic code’ of the DNA to describe the decoration of the nucleosome with posttranslational modifications (Turner, 2000); however, the so-called ‘histone code’ does not match the definition of a code, as the latter is specified by semeiotics (Turner, 2007). An important feature of the different histone modifications is that they do not take place independently from each other but are strongly interrelated in a complex, yet poorly understood manner (Margueron *et al.*, 2005). However, there are promising approaches to tackle this problem in a genome-wide or a gene-specific manner. It has just been shown very recently for the human genome that methylation and acetylation mark defined and predictive signatures on promoters and enhancers (Heintzman *et al.*, 2007). Screening along 30 Mb of the human genome revealed active promoters to be distinguished by trimethylation of K4 in histone H3, whereas the same residue in enhancers is marked by monomethylation. The methylation status of K4 in H3 will add predictive accuracy to other markers, like DNAseI-hypersensitive sites or the level of histone acetylation.

One of the first examples for the interplay of different histone modifications was the observation that phosphorylation of S10 in H3 facilitated the acetylation of H3-K9 and K14, whereas methylation of H3-K9 inhibited the phosphorylation of S10 (Rea *et al.*, 2000), although later evidence indicated a possible coexistance of phosphorylated S10 and methylated K9 (Mateescu *et al.*, 2004).

## A complex protein modification code

After the identification of the first histone acetyltransferases (HATs; Kleff *et al.*, 1995; Brownell *et al.*, 1996) and histone deacetylases (HDACs; Taunton *et al.*, 1996), it soon turned out that these enzymes not only modify histones but a huge variety of nonhistone regulatory and structural proteins that interact with chromatin, such as protooncogene products, tumor suppressor proteins, transcription factors, and enzymes (e.g. Gu & Roeder, 1997; Chan *et al.*, 2001; Vervoorts *et al.*, 2003; Zhang *et al.*, 2003c; Choi *et al.*, 2003; Meraner *et al.*, 2006). Therefore, the substrate specificity of HATs and HDACs is not restricted to histones but is expanded to a continuously growing number of nuclear, but also cytoplasmic proteins. This fact raises the question of whether the modification of histones has probably been overestimated and the equally important function of HATs and HDACs is the acetylation/deacetylation of nonhistone proteins *in vivo*. This is an important point, in that the same enzyme activities that are responsible for the acetylation/deacetylation of nucleosomal histones also acetylate/deacetylate those regulatory proteins that bind to chromatin and probably recognize the complex modification pattern established on nucleosomes. One therefore faces a currently elusive correlation between pattern formation on nucleosomes and signal establishment on histone-binding proteins. On the other hand, the posttranslational modification of regulatory proteins is controlled by additional regulatory circuits, as was shown for the acetylation of p73 by the acetyltransferase p300. Activation of p73 triggers apoptosis of tumor cells lacking functional p53 and involves the activities of c-Abl and p300. Conformational changes of p73 catalyzed by the prolyl isomerase Pin1 are crucial in this pathway. Upon treatment with chemotherapeutic drugs c-Abl enhances the phosphorylation-dependent interaction between Pin1 and p73, and this in turn promotes p73 acetylation by p300 (Mantovani *et al.*, 2004). The HAT p300 itself is methylated by a protein arginine methyltransferase resulting in modulation of cofactor activity (Xu *et al.*, 2001). These examples demonstrate that different cellular pathways are intimately interwoven and histone-modifying enzymes are part of the interface between.

Another striking result was reported recently for the p300-CBP-associated factor (PCAF); PCAF acts as a HAT that also acetylates various transcriptional regulators. In addition to its HAT activity, it also has an intrinsic ubiquitination activity that is critical for the regulation of the oncoprotein Hdm2 expression levels, and thus for p53 function (Linares *et al.*, 2007).

From the few examples mentioned above, it becomes evident that interfering with the acetylation pattern may provide a promising tool to attack diseases; indeed, HDAC inhibitors are now in clinical trials and a human application has been approved to suberoylanilide hydroxamic acid (SAHA) for the treatment of specific malignant diseases (Marks, 2007).

## Histone acetylation

A flexible and dynamic chromatin structure is essential for cells to respond and adapt to physiological and environmental changes. To meet this demand of flexibility, acetylation is a reversible process that depends on two antagonizing enzymes: HATs and HDACs. Although core histone acetylation is generally associated with active transcription, there is growing evidence that deacetylation of histones can also be responsible for the direct activation of genes (Wang *et al.*, 2002; De Nadal *et al.*, 2004). This fact, together with findings that both, HATs and HDACs, can also acetylate/deacetylate a large number of nonhistone proteins, such as transcription factors and structural proteins (see above), has complicated the elucidation of the biological effects of these enzymes (Glozak *et al.*, 2005).

## HATs

HATs catalyze the transfer of acetyl groups from acetyl-CoA to defined lysine residues of histones. Lysine site specificity thereby largely depends on the corresponding type of enzyme (e.g. Kölle *et al.*, 1998). HATs are divided into several families; however, functional and structural analysis has been focused on members of the two main groups: the GNAT and the MYST enzymes.

GCN5 of *Saccharomyces cerevisiae*, a founding member of the GNAT family, and ESA1, a MYST-type enzyme, share similar catalytic domains flanked by variable N- and C-terminal regions responsible for lysine site specificity. Although structurally related, the mechanisms by which these proteins select and acetylate core-histones are entirely different. In the MYST HATs, the acetyl group of acetyl-CoA is transferred to an active site cysteine, followed by dislocation of CoA and transfer of the acetyl moiety to the histone substrate (Yan *et al.*, 2002). In contrast, GCN5 type enzymes obey a ternary complex mechanism in which acetyl-CoA and the histone substrate sequentially bind the enzyme, followed by nucleophilic attack of the lysine ɛ-amine on the thioester of acetyl-CoA (Tanner *et al.*, 2000). However, as many other histone-modifying enzymes, both HATs were identified as components of large multisubunit complexes that are recruited to promoters by interaction with DNA-bound activator proteins and transcriptional coactivators (e.g. Utley *et al.*, 1998). For example, ESA1 is the catalytic component of the NuA4 multiprotein complex and seems to be targeted to promoters of ribosomal protein genes in *Saccharomyces cerevisiae* (Reid *et al.*, 2000). GCN5 serves as the catalytic subunit of different HAT complexes, such as the SAGA complex (Grant *et al.*, 1997). SAGA represents a prototype complex, in which GCN5 together with a second subunit (SPT7), forms a central domain responsible for catalytic activity (Wu *et al.*, 2004). Both proteins contain a bromodomain as the necessary motif for stable occupancy of the complex on acetylated promoter nucleosomes (Hassan *et al.*, 2002), where SAGA is involved as a coactivator in the transcription of stress-induced genes in *Saccharomyces* (Huisinga & Pugh, 2004).

Another important HAT family with coactivator activity is p300/CBP. Among cysteine-histidine-rich regions responsible for protein–protein interactions, a bromodomain is also present in p300/CBP. Members of the p300/CBP family are central control elements in differentiation, cell-cycle control, and apoptosis of multicellular organisms (e.g. Bannister & Kouzarides, 1996; Giles *et al.*, 1998).

Representatives of the TAFII250 enzymes represent a fourth HAT family with members in fungi, plants, insects, and mammals. TAFII250 is a subunit of the TFIID general transcription factor complex that is involved in mammalian cell cycle regulation (Wang & Tjian, 1994). TAFII250 proteins contain a double bromodomain that binds to mono- and di-acetylated H4 and to H3 tails, after it has been progressively acetylated by GCN5 at several lysines; thereby, TFIID associates with the TATA box to induce nucleosome sliding and consequently transcriptional activation (Agalioti *et al.*, 2002).

In filamentous fungi, interrelations between chromosome rearrangements and transcriptional regulation were extensively studied on several inducible promoters of *Aspergillus nidulans*, mainly by micrococcal nuclease and DNAse I-based mapping of chromatin organization (e.g. Muro-Pastor *et al.*, 1999, 2004; Narendja *et al.*, 1999, 2002; Mathieu *et al.*, 2005). Moreover, relevant work has also been carried out on the cellulase (*cbh2*) promoter of *Hypocrea jecorina* (anamorph *Trichoderma reesei*), where rearrangements of nucleosomes were found to be tightly linked to the regulation of the corresponding cellulase gene (Zeilinger *et al.*, 2003). Under inductive growth conditions, *cbh2* transcription is accompanied by displacement of the nucleosomes downstream of a *cbh2*- activating element (CAE) that is essential for *cbh2* expression by sophorose and cellulose. Subsequently, this nucleosomal dislocation leads to an accessibility of the TATA-box by the combined action of several protein complexes and enables an efficient transcription of *cbh2*, most probably by interacting with the Pol*II*-mediator complex (Zeilinger *et al.*, 2003). Although the majority of these investigations showed significant changes in the local chromatin organization of the corresponding promoters upon induction and repression, a direct connection between histone positioning and histone acetylation (or any other modification of histones) was not addressed.

However, a first important insight into chromatin rearrangements and histone acetylation was gained by exploiting the *prnD-prnB* bidirectional promoter of *Aspergillus nidulans* (e.g. Cubero & Scazzocchio, 1994; Gomez *et al.*, 2003). This intergenic region regulates transcription of two genes required for proline utilization: a proline oxidase (PrnD) and the major proline transporter (PrnB) of the fungus. Eight nucleosomes are positioned in this promoter region, which are depositioned under inductive conditions and partially repositioned under simultaneous carbon and nitrogen metabolite repression in the presence of an inducer (Garcia *et al.*, 2004). However, repositioning and repression of *prnB/prnD* failed, when the transcription factor CreA was mutated or unable to bind. Interestingly, a similar loss of nucleosome repositioning was observed, when cells were treated with trichostatin A (TSA), a well-known HDAC inhibitor. Nevertheless, derepression of both, *prnB* and *prnD*, was less pronounced under TSA treatment as in the *creA* mutant. The authors concluded that (1) nucleosome repositioning is necessary for full repression, (2) CreA is still able to repress the genes partially on completely open chromatin, and (3) a full repression mediated by CreA is associated with the acetylation state of histones. Although this study indicated the importance of histone modifications for the regulation of metabolites in *Aspergillus nidulans*, further investigations will be necessary to elucidate the specific role of acetylation and of the enzymes involved. HAT mutants and chromatin immunoprecipitation (ChIP) assays may provide further insight into the complex regulation of this bidirectional promoter.

Additional evidence for a specific regulatory role of histone acetylation indeed came from ChIP experiments. Grimaldi *et al.* (2006) demonstrated that in *Neurospora crassa*, lysine 14 of H3 in the light-inducible *albino-3* promoter becomes transiently acetylated after induction by blue light. Further experiments revealed that NGF-1, the GCN5-homologous HAT of *Neurospora*, is responsible for this light-induced acetylation and that the well-known photoreceptor protein WC-1 is crucial for this process. These data provide evidence for a direct link between the site-specific acetylation of histone H3 by a defined HAT (GCN5 homolog) and transcriptional regulation in a filamentous fungus.

In addition to GCN5, filamentous fungi possess multiple representatives of distinct HAT families (Borkovich *et al.*, 2004). With the exception of the examples mentioned, little is known about the biological functions of HATs in these organisms. The lack of data on HATs is remarkable, because filamentous fungi show a striking morphological complexity with respect to specialized structures for growth, reproduction, colonization, and infection and thus represent important tools for the investigation of various questions that are also relevant in higher eukaryotes (Casselton & Zolan, 2002); in contrast to yeast, for example, filamentous fungi contain complex I in their respiratory chain and some of them have a clearly discernable circadian rhythm. Moreover, the completely annotated genomes of different representatives of these organisms revealed that they possess a large number of genes without homologues in *Saccharomyces cerevisae* (Osiewacz, 2002). These facts, together with the rapid development of molecular and genetic techniques for filamentous fungi, have made species like *Neurospora, Ascobolus*, and *Aspergillus* model organisms that contributed significantly to an understanding of basic biological phenomena.

Another fascinating aspect of filamentous fungi is the production of SM, which are considered to be part of the chemical arsenal required for niche specialization of these organisms. SMs have attracted attention by virtue of their biotechnological and pharmaceutical applications (Demain & Fang, 2000; Calvo *et al.*, 2002). Many fungi display a broad range of useful antibiotic, antiviral, antiproliferative, antihypercholesterolemic, and immunosuppressive activities, but also phyto- and mycotoxic activities that make them important items for human and veterinary medicine, biotechnology, and agriculture.

Among these fungal products are potent HDAC inhibitors, the well-known antibiotic penicillin, as well as the carcinogen aflatoxin (e.g. Brakhage, 1998; Graessle *et al.*, 2001; Bok & Keller, 2004). Thus, elucidation of SM expression in fungi is of substantial scientific, economic, and medical relevance. Recently, a strong connection was demonstrated between the regulation of certain SMs, histone modifications, and HATs (Keller *et al.*, 2006; Roze *et al.*, 2007; Shwab *et al.*, 2007).

## HDACs

HDACs catalyze the removal of acetyl groups from lysine residues of core histone tails; however, many of these enzymes are not unique to eukaryotes, but were also found in archea and eubacteria, indicating that HDACs are an evolutionary ancient protein family that have nonhistone substrates as well (Leipe & Landsman, 1997). Today, HDACs are categorized into three families: (1) The sirtuins (Table 1); (2) the classical HDACs (Table 2); and (3) the HD2-like enzymes. The latter are exclusively found in plants (Loidl, 2004) and will therefore not be discussed here.

**Table 1:** Classical histone deacetylases in different fungal phyla

Phylum Locus, Acc. no., match	RPD3-type Locus, Acc. no., match	HOS1-type Locus, Acc. no., match	HOS2-type Locus, Acc. no., match	HOS3-type Locus, Acc. no., match	HDA1-type Locus, Acc. no., match
*Ascomycota*					
*Saccharomycotina*					
*Saccharomyces cerevisiae*	XIV, NP_014069, 0.0	XVI, NP_015393, 0.0	VII, NP_011321, 0.0	XVI, NP_015209, 0.0	XIV, NP_014377, 0.0
*Klyveromyces lactis*	E, XP_454037, 0.0	B, XP_451946, 1e-115	F, XP_455495, 0.0	F, XP_455118, 0.0	E, XP_454328, 0.0
*Pichia stipitis*	1, EAZ62851, 0.0	1, EAZ62768, 7e-60		1, EAZ63944, 4e-152	
*Yarrowia lipolytica*	E, XP_504286, 0.0	E, XP_504627, 8e-46	C, XP_501501, 2e-160	A, XP_5003054, e-113	E, XP_504372, 0.0
*Taphrinomycotina*					
*Schizosaccharomyces pombe*	II, NP_595333, 5e-149		I, NP_594079, 6e-142		II, NP_595104, 7e-147
*Pezizomycotina*					
*Aspergillus nidulans*	III, EAA60836, 4e-176		II, EAA60014, 3e-134	IV, EAA61665, 6e-81	II, EAA59664, 1e-117
*Aspergillus fumigatus*	2, XP_749474, 4e-174		2, XP_749513, 2e-135	4, XP_746614, 3e-78	5, XP_748144, 9e-94
*Neurospora crassa*	I, EAA35131, 5e-163		I, EAA35215, 7e-113	IV, EAA32603, 3e-85	II, EAA27738, 1e-132
*Basidiomycota*					
*Coprinopsis cinerea*	ND, EAU93448, 7e-135			ND, EAU86292, 3e-54	ND, EAU91781, 1e-105
	ND, EAU82749, 2e-129				
*Cryptococcus neoformans*	6, XP_571598, 4e-165		8, XP_572385, 4e-138	1, XP_566984, 2e-41	3, XP_569378, 9e-105
	8, XP_572517, 7e-132				
*Ustilago maydis*	5, EAK83185, 8e-169		1, EAK80829, 3e-121	16, EAK85982, 2e-51	5, EAK83157, 3e-94
	2, EAK81855, 6e-139				

NCBI blast searches (http://www.ncbi.nlm.nih.gov/BLAST/) were done against selected fungal genomes using the corresponding HDAC sequences of *Saccharomyces cerevisiae* as references. Chromosome (location), accession number (Acc. no.), and the E-value (match) are given.

**Table 2:** Sirtuins in different fungal phyla

Phylum Locus, Acc.no., match	SIR2-type Locus, Acc.no., match	HST1-type Locus, Acc.no., match	HST2-type Locus, Acc.no., match	HST3-type Locus, Acc.no., match	HST4-type Locus, Acc.no., match
*Ascomycota*					
*Saccharomycotina*					
*Saccharomycescerevisiae*	IV, NP_010242, 0.0	XV, NP_014573, 0.0	XVI, NP_015310, 0.0	XV, NP_014668, 0.0	IV, NP_010477, 0.0
*Klyveromyces lactis*		F, XP_455739, 2e-170	F, XP_455583, 1e-87	A, XP_451318, 1e-129	D, XP_453882, 1e-83
*Pichiastipitis*	2, XP_001382290, 8e-180		5, XP_001384758, 1e-74	7, XP_001386134, 2e-91	
	1, XP_001387128, 2e-108				
*Yarrowia lipolytica*	F, EAA59855, 2e-60	F, XP_505293, 6e-60		D, XP_502314, 2e-48	C, XP_502162, 1e-61
*Taphrinomycotina*					
*Schizosaccharomyces pombe*		II, NP_001018840, 7e-72	III, NP_588147, 8e-64		I, NP_593659, 9e-55
*Pezizomycotina*					
*Aspergillus nidulans*		II, EAA59855, 3e-64	IV, EAA62041, 2e-62		VIII, EAA65819, 2e-51
			ND, EAA67072, 1e-46		
*Aspergillus fumigatus*		4, XP_751618, 1e-81	2, XP_749719, 3e-59		1, XP_752420, 9e-52
			3, XP_748372, 1e-49		
*Neurospora crassa*		VI, EAA31136, 2e-80	I, EAA34489, 1e-59		I, EAA34475, 1e-53
*Basidiomycota*					
*Coprinopsis cinerea*	ND, EAU90220, 1e-47		ND, EAU86331, 7e-52	ND, EAU90600, 3e-56	
				ND, EAU85410, 5e-40	
*Cryptococcus neoformans*	10, XP_567573, 4e-66		7, XP_572104, 4e-61	1, XP_566762, 5e-56	
*Ustilago maydis*		2, EAK81724, 4e-66	20, EAK86837, 2e-52		

NCBI blast searches (http://www.ncbi.nlm.nih.gov/BLAST/) were done against selected fungal genomes using the corresponding sirtuin (HST) sequences of *Saccharomyces cerevisiae* as references. Chromosome (location), accession number (Acc. no.), and the E-value (match) are given. Note, that only those sirtuins were included in the table, that are closely related to the four yeast HST types.

### The sirtuins

Sirtuins are NAD^+^-dependent HDACs with sequence homology to the silent information *r* egulator (SIR2) protein from *Saccharomyces cerevisiae*. Initially identified as phosphoribosyltransferases in bacteria (Tsang & Escalante-Semerena, 1998), SIR2-type proteins possess HDAC-activity as well (Imai *et al.*, 2000; Landry *et al.*, 2000; Smith *et al.*, 2000). In addition to SIR2, four other homologous SIR two proteins (HST1-HST4) were found in the genome of *Saccharomyces cerevisiae* (Table 2), with at least one ortholog in almost all organisms examined so far (Frye, 2000). As expected, sequences of several putative SIR2-type proteins are also present in the genomes of filamentous fungi (Borkovich *et al.*, 2004), but little is known about the catalytic activity, the specificity, and the biological functions of these proteins in fungi. However, a significant proportion of total HDAC activity in whole-cell extracts of *Aspergillus nidulans* could be assigned to an NAD^+^-dependent activity (Trojer *et al.*, 2003).

The biological function of SIR2 has been studied intensively in *Saccharomyces cerevisiae*, where it is involved in the silencing of Pol I-transcribed rRNA gene as part of the nucleolar RENT (regulator of nucleolar silencing and telophase exit) complex (Straight *et al.*, 1999). Deletion of *SIR2* reduces lifespan whereas overexpression elevates the longevity of *Saccharomyces* as well as that of other eukaryotes (Kaeberlein *et al.*, 1999; Tissenbaum & Guarente, 2001; Astrom *et al.*, 2003). Although the reason for the impact of SIR2 on aging is still under discussion, caloric restriction and a concomitant enhancement of SIR2-dependent rRNA gene silencing play essential roles in the longevity of cells (Jiang *et al.*, 2000; Lin *et al.*, 2002).

In addition, SIR2 is involved in another fundamental silencing process in *Saccharomyces cerevisiae*. Along with other *SIR* genes (*SIR3*, *SIR4*)*, SIR2* was identified as being essential for the repression of the *HML* and *HMR* regions, the silent mating-type loci in budding yeast (Gasser & Cockell, 2001), and the same protein complex was also found to be responsible for silencing of genes located in subtelomeric regions of chromosomes (Aparicio *et al.*, 1991).

ChIP assays, combined with microarray analysis (ChIP on CHIP) in *Schizosaccharomyces pombe*, revealed that Sir2 and a classical HDAC, Clr3, together act upon histone H3 K9/K14 throughout the genome, including the silent and subtelomeric chromosomal regions (Wiren *et al.*, 2005); this is remarkable, as it represents a functional link between two otherwise completely unrelated HDAC families: the sirtuins and the classical HDACs. Interestingly, this cross-connection between entirely different HDAC families could be confirmed by recent data from *Aspergillus nidulans*, which are discussed in detail below (Shwab *et al.*, 2007).

### Classical HDACs

Classical HDACs are currently divided into three classes: (1) the RPD3-type proteins (class 1), (2) the HDA1-type proteins (class 2), both named after the corresponding enzymes of yeast, and (3) a novel subgroup of HDACs (referred to as class 4 type enzymes) that includes HDAC11 of *Homo sapiens* and *Mus musculus*, HDA2 of *Arabidopsis thaliana*, and a representative of *Drosophila melanogaster*. Examination of the full NCBI database revealed that there are no class 4 HDACs in fungi (Gregoretti *et al.*, 2004). The fact that fungi are more closely related to animals than to plants (Lang *et al.*, 2002) supports the hypothesis that fungi have lost this HDAC class somewhere after their separation from metazoa and choanoflagellates. Functional analysis of classical HDACs of budding yeast and fission yeast revealed that most of these enzymes have nonoverlapping roles in gene repression. The principle of ‘division of labor’ between HDAC classes in the regulation of gene expression has been reviewed extensively (Kurdistani & Grunstein, 2003; Ekwall, 2005).

In *Saccharomyces cerevisiae*, five classical HDACs were identified: the class 1 enzymes RPD3, HOS1, and HOS2, and the class 2 HDACs, HDA1, and HOS3. HOS3 is only distantly related and is distinguished by sequence motifs that may be important for increased resistance against well-known HDAC inhibitors, such as TSA or HC toxin (Carmen *et al.*, 1999). Interestingly, HOS3-type proteins are fungal-specific and have been reported in *Candida* and filamentous fungi (Srikantha *et al.*, 2001; Trojer *et al.*, 2003), but interestingly do not exist in fission yeast (Ekwall, 2005).

### Functional analysis of specific HDACs in filamentous fungi

The first of the classical enzymes identified in filamentous fungi were RpdA and HosA of *Aspergillus nidulans* (Graessle *et al.*, 2000) and HDC2 and HDC1 of the plant pathogenic fungus *Cochliobolus carbonum* (Brosch *et al.*, 2001), which showed homology to *Saccharomyces cerevisiae* RPD3 and HOS2, respectively. However, despite pronounced sequence similarity in the catalytic regions, significant differences were also observed. The most remarkable is an extension of the C-terminal region of RPD3-type enzymes in filamentous fungi, representing a unique and highly conserved motif that is essential for the biological activity (Tribus & Graessle, unpublished data). The true function of the C-terminal tail is still elusive; however, there is evidence that it is essential for binding of fungal-specific complex partners that are necessary for the biological function of RpdA. Like most classical HDACs, fungal RPD3-type proteins also function as part of large protein complexes (e.g. Lechner *et al.*, 2000; Trojer *et al.*, 2003; Carrozza *et al.*, 2005) and in general are associated with gene repression. In the corn smut fungus *Ustilago maydis* for instance, an RpdA-related enzyme affects the regulation of a specific set of genes repressed during the haploid stage and is necessary for proliferation of diploid cells during teliospore development (Reichmann *et al.*, 2002); furthermore, it acts as a repressor of the biotrophic marker gene *mig1*, a small cysteine-containing, hydrophilic protein that is strongly up-regulated after fungal penetration into host epidermal maize cells (Torreblanca *et al.*, 2003).

Although mainly involved in gene repression, there is increasing evidence that members of this HDAC class may also be responsible for direct activation of genes. In *Cochliobolus carbonum* deletion of the class 1 HDAC, HDC1, led to strongly reduced virulence on maize plants as a result of a reduced expression of extracellular depolymerases, required for growth on alternative carbohydrates and the depolymerization of plant cell walls (Baidyaroy *et al.*, 2001). Because repression of glucose-regulated genes in filamentous fungi is mediated by the repressor CreA (equivalent to yeast MIG1), a first explanation for the requirement of HDC1 in expression of these genes was that HDC1 is responsible for repression of CreA. However, further investigations showed that *creA* expression was reduced, rather than enhanced, in *HDC1* mutants. Moreover, other factors involved in expression of fungal depolymerases (e.g. protein kinase SNF1) were not affected in these mutant strains (Baidyaroy *et al.*, 2001). These results finally led to the assumption that the HOS2 ortholog HDC1 may be directly involved in gene activation in *Cochliobolus*. Recently, this was further substantiated by the finding that in *Saccharomyces cerevisiae*, HOS2 preferentially binds to the coding region of independently up-regulated genes (the *GAL* cluster and *INO1*) during gene activation (Wang *et al.*, 2002). Moreover, the osmotic stress-activated MAP kinase HOG1 has been shown to recruit the RPD3-SIN3 HDAC complex and targets RPD3 to specific osmostress-responsive genes and, concomitantly, converts a protein repressor into an activator (De Nadal *et al.*, 2004).

In *Aspergillus nidulans*, a similar function can be assumed for the HDA1-type HDAC HdaA. Previously purified as a high-molecular-weight complex (∼450 kDa; Trojer *et al.*, 2003), deletion of HdaA resulted in a dramatic reduction of total HDAC activity and in significantly reduced growth on substrates, whose catabolism contributes to oxidative stress conditions in the fungus (Tribus *et al.*, 2005). Further analysis revealed that a failure in the induction of the catalase CatB is a major reason for increased sensitivity of the delta *hdaA* strains under oxidative stress conditions. Genome-wide microarray deacetylation maps of *Saccharomyces cerevisiae* confirm this suggestion; in this organism, it was demonstrated that HDA1 preferentially targets genes involved in drug transport, detoxification, and stress response (Robyr *et al.*, 2002). Reactive oxygen species (ROS) have been implicated to play an important role in the host defense against *Aspergillus fumigatus*, which is responsible for a variety of human diseases (Denning, 1998), and detoxification of ROS is probably one way to overcome the host response in these patients. Consequently, molecules responsible for detoxification can be considered to be virulence factors of the fungus (Philippe *et al.*, 2003). Because the defense systems against oxidative damage (and the associated enzyme setting) seem to be virtually identical in both *Aspergillus nidulans* and *Aspergillus fumigatus* (Paris *et al.*, 2003), a disturbance of this system by inhibition or deletion of the HdaA homologous enzyme of the pathogen could make the fungus susceptible to killing by ROS-generating phagocytes of the host organism.

In the course of these considerations, inhibitors against classical HDACs are not only a promising group of agents for the treatment of human cancer (e.g. Mai *et al.*, 2005), but may also be suitable for therapy of *Aspergillus* infections in immuno-compromised patients.

Several biologically active SMs of filamentous fungi inhibit metabolic processes of host organisms and therefore may represent the main determinants of virulence of pathogenic species. Some plant-pathogenic fungi produce potent HDAC inhibitors, like HC toxin of *Cochliobolus carbonum* (Brosch *et al.*, 1995; Graessle *et al.*, 2001). HC toxin is required for pathogenicity of *Cochliobolus* on its host plant maize (Ransom & Walton, 1997). However, this pathogenic fungus possesses histones and HDACs and thus needs means to protect itself against its own toxin. First studies on the protection mechanism against HC-toxin in *Cochliobolus carbonum* revealed that this fungus expresses a specific and highly resistant HDAC activity, apart from sensitive HDACs, and in addition to the HOS3-type enzyme, which has already been known to be less sensitive towards a variety of HDAC inhibitors. This finding led to the assumption of a resistant enzyme type that could either be a modified form of one of the well-known HDACs or a distinct and novel HDAC type (Brosch *et al.*, 2001). Another possibility of self-protection of *Cochliobolus carbonum* would be a factor that either inactivates HC-toxin or protects one of the classical HDACs from the toxic effect. Further studies supported the latter assumption because resistant *Cochliobolus* isolates seem to accumulate a substance in an age-dependent manner that interacts with sensitive HDACs; this factor was able to confer resistance not only against HC toxin but also against chemically unrelated HDAC inhibitors, such as TSA (Baidyaroy *et al.*, 2002). Interestingly, expression of this factor was dependent on *TOXE*, a transcription factor that regulates the HC-toxin biosynthetic genes, thus suggesting a link between HC-toxin production, pathogenicity, and HDAC resistance. HDAC activity of several nonpathogenic *Cochliobolus* isolates lacking HC-toxin production and saprophytic fungi, like *Aspergillus nidulans* or *Neurospora crassa*, are toxin-sensitive, indicating that resistance is not a common feature of filamentous fungi. On the other hand, HDAC activity of other fungal pathogens, like *Alternaria brassicicola* and *Diheretospora chlamydosporia*, which also produce HDAC inhibitors, is inhibitor-resistant (Baidyaroy *et al.*, 2002).

In addition to detrimental effects of SMs, low-molecular-weight fungal compounds can also be useful. Many widely used pharmaceuticals, in particular numerous antibiotics, are natural fungal products whose biosynthetic genes are found in compact clusters, most often near the telomeres of the chromosomes (Keller *et al.*, 2005). There is increasing evidence today that this local clustering of genes provides improved efficiency in a co-ordinated regulation via chromatin modifications within these subtelomeric regions. Recent data provide strong support for a role of HDACs in the suppression of clusters-derived SM production in filamentous fungi. In *Aspergillus nidulans*, deletion of HdaA (a class 2 HDAC) not only caused a decrease in catB expression but also an early and elevated gene expression of telomere-proximal small molecule clusters and finally an enhanced production of the corresponding metabolites sterigmatocystin (ST, a carcinogenic mycotoxin), its precursor norsolorinic acid (NOR), and penicillin (PN), respectively (Shwab *et al.*, 2007). The fact that flanking genes closest to these clusters were not affected in the deletion strains suggests transcriptional suppression by HdaA to be strictly localized.

Subsequent analysis of strains lacking other fungal HDAC genes (like those encoding HosB, or the SIR2-type enzyme HstA) revealed no individual effects of these enzymes on the SM clusters. However, the same deletions were characterized by significant synergistic effects in the repression of SMs in a delta hdaA background. These results are in line with early data from *Schizosaccharomyces pombe*, where both classical HDACs and sirtuins act intimately together and are required for efficient silencing of genes that are often located in subtelomeric regions (Hansen *et al.*, 2005; Wiren *et al.*, 2005).

Very recently, the impact of histone acetylation on the regulation of SM clusters in filamentous fungi was further confirmed by ChIP-analyses in *Aspergillus parasiticus* (Roze *et al.*, 2007). In this fungus, 27 genes involved in the aflatoxin biosynthesis are tightly clustered in a 70 kb region of the genome. During transcription of the aflatoxin genes, histone H4 acetylation was significantly increased in the corresponding gene promoters. Interestingly, transcriptional activation of the genes within the cluster (and also the pattern of spread of H4 acetylation) generally occurs in the same specific order, as the gene products are required in the aflatoxin pathway (Roze *et al.*, 2007).

Because production of low-molecular-weight compounds like mycotoxins (e.g. aflatoxin, sterigmatocystein, HC toxin) or medically important compounds (e.g. penicillin, lovastatin, cephalosporin) is a common phenomenon in many filamentous fungi, a closer inspection of SM regulation via chromatin-modifying enzymes in other ascomycetes is of high relevance and will have an impact on industrial production processes.

Recent investigations in *Alternaria alternata* and *Penicillium expansum* revealed an increased production of numerous unidentified SMs, when strains were treated with the HDAC inhibitor TSA (Shwab *et al.*, 2007).

Taken together, all available data suggest that HDACs function in the regulation of various SM clusters among a broad range of fungal genera and there is also evidence that histone modifications other that acetylation are involved in the regulation of fungal SM production. In *Aspergillus nidulans*, a transcription factor, LaeA, acts as a global, positively acting factor in the expression of SM clusters and appears to be a protein methyltransferase with some homology to histone methyltransferases (HMTs) (Bok & Keller, 2004). Interestingly, LaeA also exhibits a positional bias, as the transfer of genes into or out of a cluster leads to the respective gain or loss of transcription by LaeA (Bok *et al.*, 2006). The functional role of LaeA as a putative HMT will be discussed below.

The fact that deletion of LaeA in the pathogen *Aspergillus fumigatus* yields a less pathogenic strain emphasizes the impact of SM cluster regulation for fungal pathogenicity (Perrin *et al.*, 2007); further virulence analyses with *Aspergillus fumigatus hdaA* deletion strains will be of particular interest.

## Protein (de)methylation – mechanisms and enzymes

Protein methylation involves the transfer of a methyl group from *S*-adenosyl-l-methionine (SAM) to substrate proteins. Depending on the amino acid affected, lysine- and arginine-specific methylation of histones and nonhistone proteins can be discriminated. Methylation is catalyzed by two independant types of enzymes that are implicated in a number of processes including transcriptional regulation, DNA-repair, signal transduction, and protein trafficking (for a review, see Lee *et al.*, 2005a).

## Histone lysine methylation

Several lysine residues, including lysines 4, 9, 14, 27, 36, and 72 of histone H3, lysines 20 and 59 of histone H4 (Strahl *et al.*, 1999; Zhang & Reinberg, 2001; Zhang *et al.*, 2002a, b, 2003a, b), and lysine 26 of H1 (Kuzmichev *et al.*, 2004), are sites of methylation. Each lysine residue can be mono-, di-, or trimethylated by members of the SET [Su(var)3–9, Enhancer-of-zeste, Trithorax] domain-containing histone lysine methyltransferase (HKMT) family. SET domain-containing proteins are classified into at least four families, including the SUV39, SET1, SET2, and RIZ families, according to the presence or the absence and the nature of sequences surrounding the SET domains (Kouzarides, 2002). Moreover, DOT1 is a non-SET domain-containing enzyme that is important in telomeric silencing in *Saccharomyces cerevisiae* (Singer *et al.*, 1998) and humans (Feng *et al.*, 2002; Ng *et al.*, 2002).

Although histones are the predominant substrates identified for HKMTs, a few nonhistone substrates were identified previously such as Rubisco from plants (Houtz *et al.*, 1989), the tumor suppressor protein p53 (Chuikov *et al.*, 2004) and the TBP-associated factors, TAF 7 (Couture *et al.*, 2006) and TAF10 (Kouskouti *et al.*, 2004).

## HKMTs in fungi

**Fig. 1:**
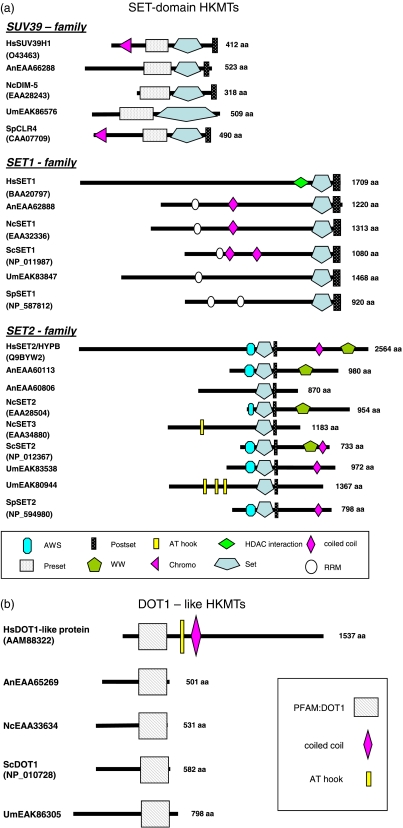

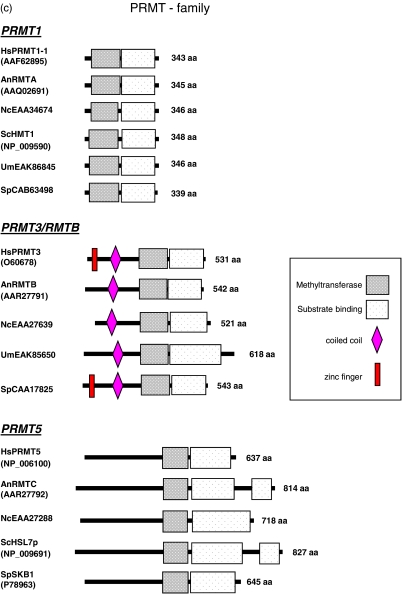

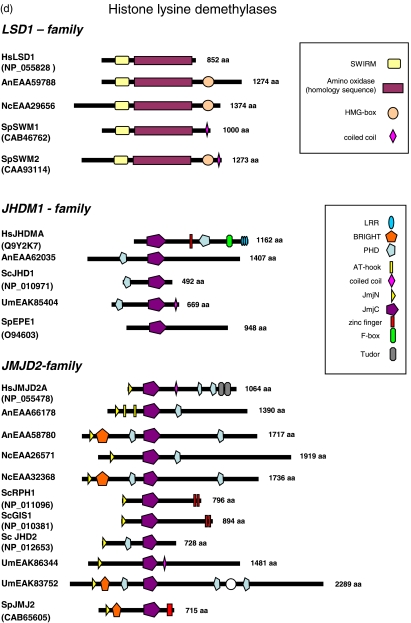
Domain architecture of human and fungal histone lysine methyltransferases (a, b), protein arginine methyltransferases (c), and histone lysine demethylases (d), respectively. The NCBI blast program (http://www.ncbi.nlm.nih.gov/blast/) was used to compare human sequences of protein family members with sequence databases of the genomes of *Aspergillus nidulans* (An), *Neurospora crassa* (Nc), *Saccharomyces cerevisiae* (Sc), *Schizosaccharomyces pombe* (Sp), and *Ustilago maydis* (Um). Sequences of human (Hs) proteins were obtained from GenBank. Domain architectures of proteins were identified and analyzed by the Simple Modular Architecture Research Tool (SMART; http://smart.embl-heidelberg.de/) and were compared with human members of each protein family. The corresponding gene IDs, number of amino acids of proteins, and accession numbers are given.

In *Neurospora crassa*, lysines 4, 27, 36, and 79 in histone H3, and lysine 20 of H4 are subject to methylation *in vivo* (Adhvaryu *et al.*, 2005). In *Neurospora* and *Aspergillus nidulans*, members of all known HKMT subfamilies, except for the RIZ family, are present (Fig. 1a). Retinoblastoma protein-Interacting Zinc finger (RIZ) proteins, such as the H3-K9 specific enzymes Meisetz (Hayashi *et al.*, 2005) or RIZ1 (Kim *et al.*, 2003), contain a conserved PR domain that is related to the SET domain. However, this domain is not found in the genomes of filamentous fungi or *Saccharomyces cerevisiae* and therefore is likely to be derived from the SET domain during evolution (Huang *et al.*, 1998).

### SUV39 family

The SUV39 family (Suppressor of Variegation H3-K9) includes the largest number of HKMTs. Members of this family methylate H3-K9 (Rea *et al.*, 2000; Schultz *et al.*, 2002; Tamaru & Selker, 2001; Selker *et al.*, 2003) or K9 and K27 of H3 (Tachibana *et al.*, 2001) and contain two cysteine-rich regions flanking the SET domain. These pre-SET and post-SET domains are required for HKMT activity (Rea *et al.*, 2000).

Like the human enzyme SUV39H1 (O43463), Dim-5 (Defective in methylation 5; EAA28243) of *Neurospora* is an H3-K9-specific HKMT that is essential for DNA methylation *in vivo* (Tamaru & Selker, 2001; Tamaru *et al.*, 2003). The crystal structure has been determined for Dim-5 (Zhang *et al.*, 2002b). Dim-5 complexed with *S*-adenosylhomocysteine revealed a pre-SET domain that forms a Zn_3_Cys_9_ triangular zinc cluster, a SAM-binding site in the SET domain, and a post-SET domain that might be essential for catalytic activity, resulting in sensitivity to metal chelators (Zhang *et al.*, 2003b). The Dim-5 homolog of *Aspergillus nidulans* (EAA66288) has a similar domain structure containing the 3 SET domains, although an extended N-terminal region is present (Fig. 1a).

In fission yeast, methylation of H3-K9 by Clr4 (Cryptic loci regulator 4) occurs preferentially at heterochromatin-associated regions to generate a binding site for Swi6, a homolog of *Drosophila* HP1 (Nakayama *et al.*, 2001). On the structural level, the Clr4 protein (CAA07709) has a conserved chromo- and SET domain (Fig. 1a) that is required for *in vivo* methylation activity (Nakayama *et al.*, 2001). In *Saccharomyces cerevisiae*, no SUV39H1 homologous protein is present.

### SET1 family

**Fig. 2:**
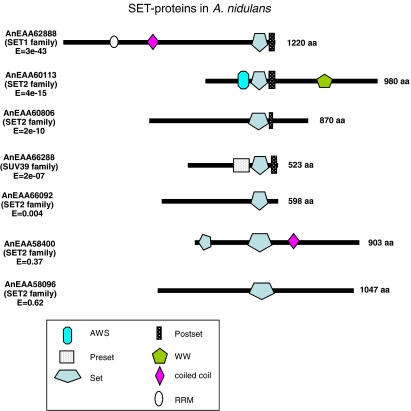
Domain structure of SET-domain proteins in *Aspergillus nidulans*. Sequences were derived by a search of the *Aspergillus nidulans* genome database (http://www.broad.mit.edu/annotation/genome/aspergillusnidulans/) for proteins containing SET domains (SM 00317), and domain structures were identified and analyzed by the Simple Modular Architecture Research Tool (SMART; http://smart.embl-heidelberg.de/). Accession numbers are provided, best-matching protein families are given in parentheses, and the number of amino acids of proteins and e-values are shown.

*Neurospora crassa* and *Aspergillus nidulans* are predicted to have nine (Adhvaryu *et al.*, 2005) and seven SET domain proteins (Fig. 2), respectively, including one hSET1 (BAA20797) homologous protein each (Fig. 1a). These proteins are characterized by the presence of three distinct regions of clear homology, an RNA recognition motif (RRM), indicating a putative role for RNA binding or protein–protein interactions (Maris *et al.*, 2005), and the SET domain, which is followed by the post-SET region. Homologous proteins in *Neurospora crassa* (EAA32336), *Aspergillus nidulans* (EAA62888), *Saccharomyces cerevisiae* (NP_011987), *Ustilago maydis* (EAK83847), and *Schizosaccharomyces pombe* (NP_587812) contain these domains, and in addition, these proteins possess one or two coiled coil domains (except for the *Schizosaccharomyces pombe* protein). Among the SET1 family proteins, budding yeast SET1 or mixed lineage leukemia (MLL) proteins are specific for H3-K4, capable of di- and trimethylation, respectively (Milne *et al.*, 2002; Santos-Rosa *et al.*, 2002), whereas EZH1/2 can methylate H3-K27 up to the trimethylated state (Cao *et al.*, 2002; Czermin *et al.*, 2002; Kuzmichev *et al.*, 2002; Muller *et al.*, 2002). Set1 of *Schizosaccharomyces pombe* also methylates H3 at K4 and, in addition, was shown to be required for efficient telomeric and centromeric silencing (Kanoh *et al.*, 2003).

### SET2 family

In the genomes of *Neurospora crassa*, *Aspergillus nidulans*, and *Ustilago maydis*, two proteins with homology to the human SET2 homolog HYPB (Huntington Yeast Partner B; Q9BYW2) are present (Fig. 1a). One of the two proteins in each organism (AnEAA60113; NcEAA28504; UmEAK83538) is characterized by an AWS (*A* ssociated *W* ith *S* ET) domain, a subdomain of the pre-SET domain, and a WW domain (with exception of the *Ustilago* protein) besides the SET and post-SET motifs. WW domains bind proline-rich polypeptides (Gao *et al.*, 2006). The *Neurospora* protein has been identified recently as an H3-K36-specific Set-2 protein that methylates H3 in transcribed genes in a nucleosome-specific way up to the trimethylated state (Adhvaryu *et al.*, 2005). Furthermore, the activity of Set-2 was essential for growth and development in *Neurospora*. It was speculated that methylation of K36 is required for the normal expression of genes involved in asexual and sexual differentiation because mutant strains displayed slow growth, sparse conidia production, and female sterility (Adhvaryu *et al.*, 2005).

Database searches further revealed that the genomes of *Neurospora crassa*, *Aspergillus nidulans*, and *Ustilago maydis* contain a second putative SET2-type HMT (AnEAA60806; NcEAA34880; UmEAK80944) lacking the AWS and WW domains (Fig. 1a).

In *Schizosaccharomyces pombe*, a single SET2-type HMT (NP_594980) is present, featuring AWS, SET, and post-SET domains and a coiled coil motif (Fig. 1a). Similar to the *Neurospora* protein, fission yeast Set2 has been shown to be a nucleosome-selective H3 methyltransferase. The finding that H3-K36 methylation by Set2 was associated with transcribed regions of Pol II-regulated genes implied a conserved role for this modification in the transcription elongation process (Morris *et al.*, 2005).

Finally, the SET2 protein of *Saccharomyces cerevisiae* (NP_012367) also exhibited specificity for H3-K36, a methylation event related to transcriptional activation (Strahl *et al.*, 2002).

### *Dot1* family

The DOT1 family includes HKMTs lacking the SET domain. The only known member of this family, DOT1 (Disrupter Of Telomere silencing 1), methylates H3-K79 (Lacoste *et al.*, 2002; van Leeuwen *et al.*, 2002), a residue that is located in the core domain of H3. In *Saccharomyces cerevisiae*, DOT1 is essential for silencing of genes near telomeres, the rRNA gene loci, and the mating-type loci (Singer *et al.*, 1998). *Neurospora crassa* (EAA33634), *Aspergillus nidulans* (EAA65269), *Saccharomyces cerevisiae* (NP_010728), and *Ustilago maydis* (EAK86305) contain a single homolog of the human DOT1-like protein (AAM88322), each with a similar domain structure (Fig. 1b). However, in contrast to the human homolog, the conserved core domain of fungal DOT1 proteins is located in the C-terminus but not in the N-terminal part. Remarkably, no DOT1 homologous protein is present in *Schizosaccharomyces pombe*.

## Histone arginine methylation

Besides lysines, arginine residues of histones can also be methylated by members of the protein arginine methyltransferase (PRMT) family (Gary & Clarke, 1998). PRMTs catalyze the transfer of methyl groups from SAM to the guanidino nitrogens of arginine. This modification occurs within the N-terminal tails of H3 (R2, R8, R17, R26) and H4 (R3). Arginine can be mono- or dimethylated, the latter in a symmetric or an asymmetric way. Enzymes have been divided into two major classes. (1) Type I enzymes, catalyzing formation of NG-monomethylarginine and asymmetric NG,NG-dimethylarginine residues, and (2) Type II enzymes, catalyzing formation of NG-monomethylarginine and symmetric NG,N′G-dimethylarginine residues. The PRMT family currently includes nine mammalian enzymes. PRMT1 (Strahl & Allis, 2000; Wang *et al.*, 2001) and *Saccharomyces cerevisiae* RMT1/HMT1 (Gary *et al.*, 1996; Henry & Silver, 1996), PRMT2 (Katsanis *et al.*, 1997), PRMT3 (Tang *et al.*, 1998), PRMT4/CARM1 (Chen *et al.*, 1999), PRMT6 (Frankel *et al.*, 2002), and PRMT8 (Lee *et al.*, 2005b) are classified as class I enzymes. PRMT5/JBP1 (Gilbreth *et al.*, 1998; Pollack *et al.*, 1999; Branscombe *et al.*, 2001), PRMT7 (Miranda *et al.*, 2004), and PRMT9 (Cook *et al.*, 2006) are class II enzymes. Arginine methylation has been observed on a variety of proteins other than histones, including transcriptional activators and coactivators, and RNA-binding proteins involved in RNA processing, transport, and stability (Krause *et al.*, 2007).

## PRMTs in fungi

Three genes encoding for PRMTs have been identified in the *Aspergillus* and *Neurospora* genomes (Borkovich *et al.*, 2004; Trojer *et al.*, 2004). In *Aspergillus*, two of these proteins, RmtA (Arginine methyltransferase A; AAQ02691) and RmtC (AAR27792), revealed sequence homology to human PRMT1 (AAF62895) and PRMT5 (NP_006100), respectively (Fig. 1c). PRMT1 is the predominant type I enzyme in mammalian cells, accounting for more than 80% of total PRMT activity (Tang *et al.*, 2000), and representatives have also been identified in fungi, plants, and animals (Krause *et al.*, 2007). PRMT1 and PRMT5 have been reported to be enzymatically active. In *Aspergillus nidulans*, both native and recombinant RmtA were specific for H4-R3 and recombinant RmtC-methylated H4 and H2A (Trojer *et al.*, 2004). The third enzyme found in *Aspergillus nidulans*, RmtB (AAR27791), revealed sequence similarities to human PRMT3 (O60678), but exhibited structural differences and a different substrate specificity and was therefore regarded as an enzyme unique to filamentous fungi (Trojer *et al.*, 2004). Consistent with these results, in a comprehensive phylogenetic study of known PRMTs, fungal RmtB homologs appeared as an outgroup of animal PRMT3 proteins (Krause *et al.*, 2007). Interestingly, filamentous ascomycetes do not have homologs of PRMT4/CARM1, an enzyme that methylates H3-R2, R17, and R26 (Schurter *et al.*, 2001; Daujat *et al.*, 2002). However, a blast search revealed a putative PRMT4/CARM1 homolog in *Ustilago maydis*. In *Saccharomyces cerevisiae*, 2 members of the PRMT family are present (Fig. 1c): the type1 HMT1 (Heterogeneous Nuclear Ribonucleoprotein Methyltransferase; NP_009590), which is related to human PRMT1 (Gary *et al.*, 1996; Henry & Silver, 1996), and HSL7 (Histone Synthetic Lethal 7; NP_009691), a homolog of human PRMT5 with specificity for H2A and H4 and bovine myelin basic protein (Lee *et al.*, 2000; Miranda *et al.*, 2006). Searches of the *Schizosaccharomyces pombe* database revealed the existence of three proteins with homology to human PRMT1 (CAB63498), PRMT3 (CAA17825), and PRMT5 (Skb1; P78963), respectively.

For Skb1 (Shk1 kinase-binding protein 1), a putative role in the control of cell polarity was reported. The authors demonstrated that direct association of Skb1 with Orb6, a kinase that is involved in the regulation of cell morphogenesis and cell cycle control, was required for the correct subcellular localization of Orb6 (Wiley *et al.*, 2003).

Arginine methylation might also be involved in the regulation of the nuclear poly(A)-binding protein Pab2 in *Schizosaccharomyces pombe* because oligomerization levels of Pab2 were influenced by RMT1-dependent methylation (Perreault *et al.*, 2007).

## Histone demethylation

For a long time, methylation was considered to be a stable and irreversible epigenetic mark that committed chromatin to a specific transcriptional state. However, the identification of the first histone demethylases has shown that histone methylation is reversible and dynamic. Three distinct classes of enzymes that antagonize histone methylation have been characterized so far.

### Peptidyl arginine deiminases

Protein arginine demethylation has been described as a deimination process that converts arginine to citrulline and methylammonium. Human peptidyl arginine deiminase 4 (PADI4/PAD4) specifically deiminates R2, R8, R17, and R26 of H3 and R3 of H4 in unmodified and monomethylated states (Cuthbert *et al.*, 2004; Wang *et al.*, 2004) and functions as a transcriptional repressor of the estrogen-signaling pathway (Wang *et al.*, 2004). Therefore, consistent with the proposed function of arginine methylation, PAD4 antagonizes transcriptional activation by active deimination.

### Lysine-specific demethylase 1 (LSD1)

The first histone lysine demethylase was identified by Shi *et al.* (2004). LSD1 demethylates H3-K4. The enzyme cannot cleave the N–CH_3_ bond directly but induces amine oxidation of methylated H3-K4 to generate unmodified lysine and formaldehyde in an FAD-dependent manner. Only mono- or dimethylated but not trimethylated H3-K4 is subject to demethylation. LSD1 functions as a transcriptional corepressor participating in the silencing of endogenous neuron-specific genes (Lunyak *et al.*, 2002; Shi *et al.*, 2004). In addition to its repressor function, LSD1 was shown to stimulate androgen-receptor-dependent transcription; upon binding to nuclear androgen hormone receptor, LSD1 changed its substrate specificity from H3-K4 to H3-K9 (Metzger *et al.*, 2005).

### JmjC domain containing histone demethylases

Recently, Jumonji C (JmjC) domain-containing proteins have been identified as enzymes demethylating histone lysine residues. JmjC domain proteins are predicted to be hydroxylases and are classified into several subclasses (Klose *et al.*, 2006a). Using a biochemical approach, JHDM1A (JmjC Domain-Containing Histone Demethylase 1 A) was purified and reported to demethylate H3-K36. This reaction occurs in the presence of Fe^2+^ and α-ketoglutarate and generates formaldehyde and succinate; the JmjC domain was also identified as the catalytic moiety involved in mediating the demethylation reaction (Tsukada *et al.*, 2006).

JHDM2A, a member of the JHDM2 family, associates with the androgen receptor and is important for H3-K9 demethylation during ligand-dependent activation of androgen-responsive genes (Yamane *et al.*, 2006).

Finally, JHDM3/JMJD2 histone demethylases target H3-K9 and H3K-36 and are capable of removing the trimethyl modification. They are implicated in the regulation of gene expression (Klose *et al.* 2006b) and can antagonize pericentric trimethylated H3-K9 (Fodor *et al.*, 2006). Recent evidence in different organisms demonstrated that members of the JMJC family demethylate H3-K4, which is a mark of transcriptionally active chromatin (Metzger & Schüle, 2007).

Very recently, a Jumonji domain protein revealed arginine-specific demethylase activity; Bruick and colleagues demonstrated that JMJD6, a JmjC-containing iron- and 2-oxoglutarate-dependent dioxygenase, demethylates histone H3 at arginine 2 and histone H4 at arginine 3 in both biochemical and cell-based assays (Chang *et al.*, 2007).

## Histone demethylases in fungi

### PAD4

So far, PAD4/PADI4 homologous proteins have only been found in mammals. A search for fungal PADI4 homologs and for dimethylarginine dimethylaminohydrolases, enzymes that also act on free methyl-arginine, revealed no significant similarities (E-values higher than 0.05). Therefore, deimination may not play a role in fungi, and other mechanisms/enzymes for the removal of methyl groups might exist in these organisms.

### LSD1

LSD1 homologs have a SWIRM domain (Swi3p, Rsc8p, and Moira), which is present in a number of chromatin-associated proteins (Aravind & Iyer, 2002), and a long C–terminal domain with sequence homology to FAD-dependent amine oxidases.

In *Neurospora crassa* (EAA29656) and *Aspergillus nidulans* (EAA59788), one striking homolog of LSD1 can be found (1e-58 and 1e-66, respectively; Fig. 1d). The spcificity of these putative demethylases has not yet been determined. However, methylation of H3-K4 in *Neurospora* has been reported; thus, the LSD1 homolog in filamentous fungi might have a similar specificity as observed for the human isoform (Tamaru *et al.*, 2003; Adhvaryu *et al.*, 2005). Interestingly, *Aspergillus* and *Neurospora* homologs exhibit an extended C-terminal region containing an HMG box (Fig. 1d) in addition to the SWIRM domain and the amine oxidase homology sequence. The presence of HMG-box domains in filamentous fungi might indicate a divergent role/regulation of these enzymes. *Saccharomyces cerevisiae* and *Ustilago maydis* genomes contain no obvious LSD-1 homologs, although they do encompass many putative amine oxidases. In contrast, *Schizosaccharomyces pombe* contains two predicted LSD1 family members (Swm1, CAB46762, and Swm2, CAA93114). Whereas Swm2 has a domain structure similar to the *Neurospora* or *Aspergillus* proteins with an additional coiled coil motif at the C-terminus, Swm1, as the mammalian enzyme, lacks an HMG domain (Fig. 1d). Swm1 and Swm2 (Swirm1, Swirm2) are present in a protein complex with specificity for lysine 9 in histone H3. Chromatin-immunoprecipitation-coupled DNA microarray analysis suggested an important role of Swm1 in the regulation of heterochromatin propagation as well as for transcription of a large number of genes by its demethylase activity (Lan *et al.*, 2007; Opel *et al.*, 2007).

### JmjC domain demethylases: JHDM1/FBX11

Mammalian homologs of the JHDM1 group contain an F-box, leucine-rich repeats (LRRs), and CXXC zinc-finger domains, in addition to the JmjC domain. The F-box domain was first described as a sequence motif found in cyclin-F that interacts with the protein SKP1 (S-phase Kinase-associated Protein 1) to form the SCF complex (Skp1-Cullin-F-box). Different F-box proteins as part of the SCF complex recruit particular substrates for ubiquitination through specific protein–protein interaction domains (Bai *et al.*, 1996). Thus, F-box domains serve as recognition motifs for ubiquitination targets, suggesting that JHDM1A might link histone demethylation to protein ubiquitination.

In the blast search, JHDM1A homologs were found for *Aspergillus nidulans* (1e-57; EAA62035), *Ustilago maydis* (1e-52; EAK85404) *Saccharomyces cerevisiae* (1e-44, Jhd1, NP_010971), and *Schizosaccharomyces pombe* (1e-42, Epe1, O94603). Except for *Schizosaccharomyces pombe* Epe1, an enzyme that has been shown recently to be involved in the modulation of heterochromatin (Ayoub *et al.*, 2003; Isaac *et al.*, 2007), the other related proteins have, in addition to the shared JmjC domain, domains that imply DNA-binding and/or protein–protein interaction including N-terminal PHD zinc finger or coiled coil motifs but no F-box (Fig. 1d). Surprisingly, homologs of JHDM1A are apparently not present in *Neurospora*.

### JmjC domain demethylases: JMJD2/JHDM3A

In higher eukaryotes, proteins of this family contain JmjN, PHD, and Tudor domains in addition to the JmjC domain. The Tudor domain is absent in JMJD2/JHDM3A homologs of filamentous fungi, *Saccharomyces cerevisiae* and *Schizosaccharomyces pombe*. Tudor domains are a major class of methyl-binding domains that interact with methyllysine marks (Kim *et al.*, 2006) and methylated arginines (Friesen *et al.*, 2001; Cheng *et al.*, 2007). Recently, a role of the tandem Tudor domain of JHDM3A/JMJD2A has been assigned to a putative chromatin targeting module that may directly bind methylated H3-K4 and K9 and H4-K20 (Huang *et al.*, 2006; Kim *et al.*, 2006).

In filamentous fungi, two homologs of JMJD2/JHDM3A are present, which differ in the extent of additional PHD and HTH (Helix Turn Helix) motifs (Fig. 1d). PHD (Plant Homeodomain) fingers are C4HC3 zinc-finger-like motifs in nuclear proteins thought to be involved in epigenetics and chromatin-mediated transcriptional regulation (Mellor, 2006). Whereas one of the two proteins has an additional PHD domain (AnEAA66178; NcEAA26571), the second protein contains two PHD domains and one HTH motif (BRIGHT domain) in addition to the two Jumonji domains (AnEAA58780; NcEAA32368). *Saccharomyces cerevisiae* has three homologs of the JMJD2/JHDM3 family; two of them (RPH1, NP_011096 and GIS1, NP_010381) have two zinc finger domains, whereas the third homolog (JHD2, NP_012653) has a PHD domain. For RPH1, specific demethylation of H3-K36me3 and K36me2 was demonstrated and this activity directly regulated K36 methylation in transcribed regions, indicating a function of this enzyme in the process of transcriptional elongation by antagonizing repressive Lys(36) methylation by SET2 (Kim & Buratowski, 2007).

*Ustilago maydis* (EAK86344) has two homologs, the first revealing JmjN and JmJC domains, whereas the second predicted histone demethylase (EAK83752) has a complex domain structure with two PHD domains, HTH and RING-finger motifs, as well as JmjN/JmjC domains. Recently, Shi and coworkers have demonstrated H3-K4me3 specificity for the fission yeast enzyme Jmj2 (CAB65605) and they showed that the demethylase activity of this enzyme was necessary for the regulation of heterochromatin at the mating-type locus. Therefore, the authors suggested a putative function of this enzyme in heterochromatin function (Huarte *et al.*, 2007). Jmj2, the shortest protein among the JMJD2 family analyzed in this study, has a BRIGHT domain and a zinc finger motif besides the JmjN/JmjC domains (Fig. 1d).

### JmjC domain demethylases: JHDM2A/B/C

The JHDM2 family members possess JmjC and modified zinc finger domains and have homologs from flies to humans but are not present in fungi (Klose *et al.* 2006a).

### Comparative analysis of *Aspergillus* species

As a remarkable feature, the genus *Aspergillus* contains an astonishing variety of species that are on the one hand, harmful and, on the other, beneficial to humans with a large number of species that are of biomedical and industrial significance. *Aspergillus flavus* is a plant and animal pathogen that also produces the potent carcinogen aflatoxin (Hedayati *et al.*, 2007), whereas several other species (e.g. *Aspergillus fumigatus* and *Aspergillus terreus*) are important opportunistic pathogens of individuals with compromised immune systems (Brakhage & Langfelder, 2002). In contrast, *Aspergillus niger* is widely exploited by the fermentation industry for the production of citric acid (Schuster *et al.*, 2002), whereas *Aspergillus oryzae* plays a key role in the fermentation process of several traditional Japanese beverages and sauces (Abe *et al.*, 2006). Finally, *Aspergillus nidulans* is a key fungal model system for genetics and cell biology (Casselton & Zolan, 2002).

**Fig. 3:**
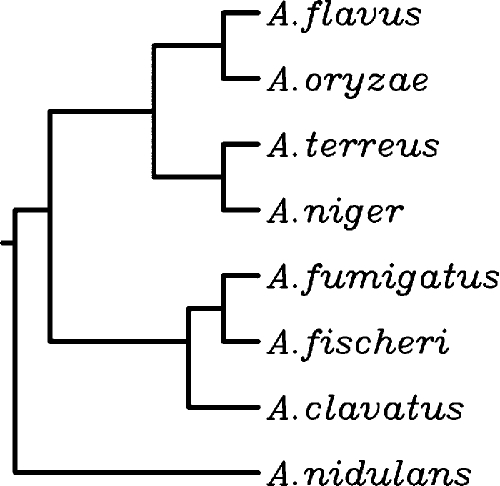
Phylogenetic relationships within the genus *Aspergillus*. SUV39H1 homologous proteins in *Aspergillus* were searched using the *Aspergillus* Comparative Database from the BROAD Institute (http://www.broad.mit.edu/). Corresponding proteins were identified by a blast search (http://www.ncbi.nlm.nih.gov/blast/) and Multiple Sequence Alignment and building of the dendrogram was performed by the clustalw program (http://clustalw.genome.jp/).

So far, the genome sequence of as many as nine *Aspergillus* species has been partially or fully completed (Jones, 2007). The sequences of *Aspergillus nidulans*, *Aspergillus fumigatus*, *Aspergillus oryzae*, *Aspergillus niger*, *Aspergillus clavatus*, *Aspergillus flavus*, *Aspergillus terreus*, and *Aspergillus fischerianus* are available for the scientific community; sequencing of the genome of *Aspergillus parasiticus* is in progress. The great importance of the genus *Aspergillus* for pathogenicity, industrial applications, and scientific issues and the availability of a variety of genome sequences prompted the analysis of conservation, variation, and distribution of protein (de)methylating enzymes in eight closely related *Aspergillus* species. Genome statistics of analyzed species are shown in Table 3. Accession numbers of proteins and the total number of obtained matches are given in Tables 4 and 5. Furthermore, a phylogenetic analysis within the genus *Aspergillus* was performed on the basis of homologs of human SUV39H1 protein. The dendrogram shown in Fig. 3 thereby displays similar phylogenetic relationships of the different *Aspergillus* species as demonstrated recently for a genome-scale analysis (Rokas & Galagan, 2007). Note that similar phylogenies were also obtained when other histone-modifying enzymes, e.g. homologs of Rpd3, were used in the phylogenetic studies (data not shown).

**Table 3:** Genome statisitics of different *Aspergillus* species (Rokas & Galagan, 2007)

*Aspergillus* species	Size	Chromosomes	Genes
*A. fumigatus*	29.385 Mb	8	9887
*A. flavus*	36.790 Mb	8	12 604
*A. nidulans*	30.069 Mb	8	10 701
*A. niger*	37.196 Mb	8	11 200
*A. terreus*	29.331 Mb	8	10 406
*A. oryzae*	37.118 Mb	8	12 336
*A. fischerianus*	32.552 Mb	8	10 406
*A. clavatus*	27.859 Mb	8	9121

**Table 4:** Presence of histone lysine methyltransferases and protein arginine methyltransferases in different *Aspergillus* species

	Protein methyltransferases						
	
	SET-domain HKMTs			DOT1-like HKMTs	PRMT-family		
			
	SUV39	SET1	SET2	DOT1	PRMT1	PRMT3/RMTB	PRMT5
*A. nidulans*	1 EAA66288	1 EAA62888	2 EAA60113 EAA60806	1 EAA65269	1 AAQ02691	1 AAR27791	1 AAR27792
*A. clavatus*	1 XP_001269157	1 XP_001272547	2 XP_001273933 XP_001270666	1 XP_001274501	1 XP_001269526	1 XP_001271243	1 XP_001274457
*A. terreus*	1 XP_001210396	1 XP_001216024	2 XP_001218053 XP_001217362	1 XP_001211240	1 XP_001212165	1 XP_001213321	1 XP_001211288
*A. niger*	1 XP_001392454	No match	2 XP_001398411 XP_001394944	1 XP_001393586	1 XP_001398973	No match	No match
*A. oryzae*	1 BAE64107	1 Q2UMH3	2 Q2UTN6 BAE63337/BAE63336	1 Q2U696	1 BAE56950	1 BAE55751	1 BAE60082
*A. flavus*	1 AFL2G_07659.2	1 AFL2G_02936.2	2 AFL2G_10072.2 AFL2G_11178.2	1 AFL2G_08335.2	1 AFL2G_09078.2	1 AFL2G_00708.2	1 AFL2G_06640.2
*A. fumigatus*	1 XP_752474	1 XP_750524	2 XP_754032 XP_750524	1 XP_753440	1 XP_750368	1 XP_754372	1 XP_753489
*A. fischerianus*	1 XP_001264660	1 XP_001257747	2 XP_001266036 XP_001263805	1 XP_001259486	1 XP_001265048	1 XP_001263214	1 XP_001259533

Number of homologs and accession numbers are indicated. The genome of *Aspergillusflavus* has not been published yet, therefore the gene locus derived from the comparative database search (BROAD) is indicated.

**Table 5:** Presence of histone demethylases in different *Aspergillus* species

	Histone lysine demethylases		
	
	LSD1 – family	JHDM1 – family	JMJD2 – family
*A. nidulans*	1 EAA59788	1 EAA62035	2 EAA66178 EAA58780
*A. clavatus*	1 XP_001272154	1 XP_001275235	2 XP_001269026 XP_001276189
*A. terreus*	1 XP_001212360	1 XP_001215965	2 XP_001210517 XP_001216692
*A. niger*	1 XP_001389280	1 XP_001400562	2 XP_001392614 XP_001393891
*A. oryzae*	1 BAE54767	1 BAE57204	2 BAE56833 BAE61572
*A. flavus*	1 AFL2G_10384.2	1 AFL2G_09344.2	2 AFL2G_07522.2 AFL2G_09928.2
*A. fumigatus*	1 XP_751531	1 XP_749726	2 CAD27311 XP_748000
*A. fischerianus*	1 XP_001266744	1 XP_001260218	2 XP_001264528 XP_001266191

Number of homologs and accession numbers are indicated. The genome of *Aspergillusflavus* has not been published yet, therefore the gene locus derived from the comparative database search (BROAD) is indicated.

### Protein methyltransferases and demethylases in *Aspergillus* species

**Fig. 4:**
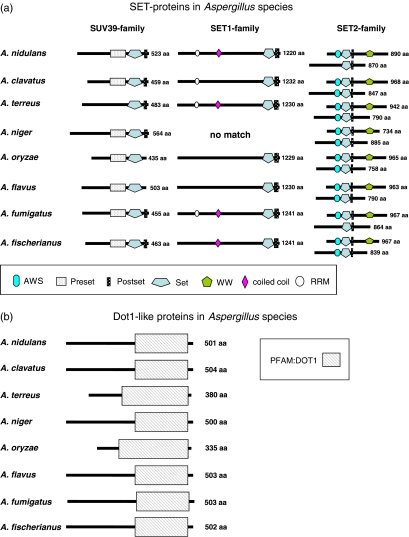

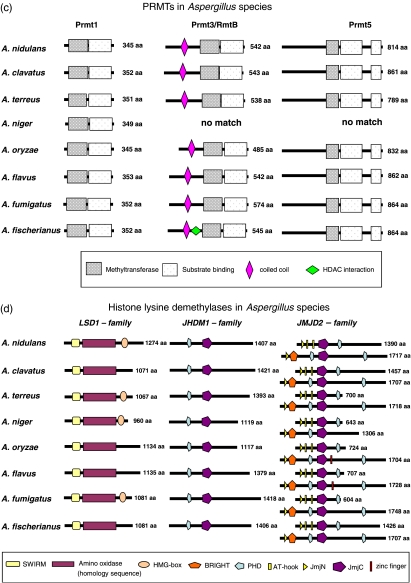
Domain architecture of histone lysine methyltransferases (a, b), protein arginine methyltransferases (c), and histone demethylases (d) of different *Aspergillus* species. *Aspergillus* homologs of human proteins (see Fig. 1) were searched using the *Aspergillus* Comparative Database from the BROAD Institute (http://www.broad.mit.edu/). Corresponding proteins were identified by a blast search (http://www.ncbi.nlm.nih.gov/blast/) and domain architectures of proteins were analyzed by the Simple Modular Architecture Research Tool (SMART; http://smart.embl-heidelberg.de/). The number of amino acids of proteins is shown. Accession numbers are given in Tables 4 and 5.

*SET-proteins*. All analyzed *Aspergillus* species have a single SUV39 or SET1 homolog except for *Aspergillus niger*, where apparently no SET1 homologous protein is present as determined by a database search (Fig. 4a, Table 4). Moreover, three of the eight SET1 proteins (*Aspergillus oryzae, Aspergillus flavus*, and *Aspergillus fischerianus*) do not contain an RNA recognition motif (RRM) that was found in homologs of other fungal genomes (compare Fig. 1a). All organisms contain two SET2 homologs with high conservation of the domain structure compared with the *Aspergillus nidulans* or *Neurospora crassa* enzymes.

*DOT1-proteins*. In all *Aspergillus* species, one gene encoding a putative DOT1 homologous protein is present. Remarkably, in *Aspergillus terreus* and *Aspergillus oryzae* the primary sequence of the corresponding proteins is reduced in size and is restricted to cover mainly the conserved DOT1 domain (Fig. 4b).

*PRMT proteins*. The initial search for PRMT homologs in selected eukaryotic organisms (see Fig. 1a) had revealed that in filamentous fungi, three enzymes are present: two type I PRMTs and one type II PRMT. This result could be confirmed for the individual *Aspergillus* species with one remarkable exception: the *Aspergillus niger* genome contains only one PRMT homologous protein, no match could be obtained either for the PRMT3/RmtB group or for PRMT5. This is a striking finding because in most organisms investigated so far, at least one type I and one type II enzyme is present (Krause *et al.*, 2007). All RmtB homologs contain a coiled coil motif but lack the zink finger domain that was identified in human PRMT3. Moreover, for *Aspergillus fischerianus*, the SMART analysis indicated the presence of an HDAC domain, a motif found on transcriptional regulators that forms interactions with HDACs.

### Demethylases

Genomes of all tested *Aspergillus* species include one LSD1 homolog that exhibits variations in the domain structure; in *Aspergillus oryzae, Aspergillus clavatus, Aspergillus flavus*, and *Aspergillus fischerianus*, an HMG box is missing, which is one of the characteristic features of other fungal LSD1 homologs (compare Fig. 1d). Importantly, the HMG motif is embedded in a 50 aa sequence stretch that shows only little homology between the different species (data not shown). Besides LSD1 homologs, further *Aspergillus* genes that are predicted to encode amine oxidases were identified. In *Aspergillus nidulans, Aspergillus clavatus* two genes, in *Aspergillus fumigatus, Aspergillus oryzae, Aspergillus terreus*, and *Aspergillus fischerianus* three genes, and in *Aspergillus niger* and *Aspergillus flavus* four further genes are present (data not shown).

Within the class of JmjC domain containing histone demethylases, three homologs to human enzymes are present. All members of the JHDM1-family are uniform in offering JmjC and PHD domains. With respect to the JMJD2-family, the search revealed two matches for each *Aspergillus* species. The AT-hook containing enzyme forms thereby differed considerably in the length of the overall protein. Thus, the sequences of the corresponding proteins in *Aspergillus fumigatus, Aspergillus oryzae, Aspergillus niger, Aspergillus flavus*, and *Aspergillus terreus* have only half the size (from 604 to 724 aa) compared with proteins of *Aspergillus nidulans, Aspergillus clavatus*, and *Aspergillus fischerianus* (from 1390 to 1457 aa). Nevertheless, all identified structural motifs including the JmjN, JmjC, PHD, and the AT-hook domains are present in all variations of proteins (Fig. 4d).

## Protein methylation – functional implications

### Control of DNA methylation by HMTs

A landmark discovery has been made in the filamentous fungus *Neurospora crassa*. In this organism, histone lysine methylation has a direct effect on the epigenetic modification of DNA. By a screen of methylation mutants in *Neurospora*, an HMT was identified and determined to be necessary for DNA methylation and gene silencing (Tamaru & Selker, 2001). Dim-5, which is related to the SET-domain containing proteins Clr4 (Ivanova *et al.*, 1998) and SU(VAR)3-9 (Tschiersch *et al.*, 1994), showed specificity for H3-K9, a position that is of particular significance for gene silencing during heterochromatin formation. Importantly, methylated DNA regions in *Neurospora* are marked by trimethylation of H3-K9 (Tamaru *et al.*, 2003; Zhang *et al.*, 2003b). Furthermore, substitutions of H3-K9 caused hypomethylation of DNA, thus indicating a close interdependence of DNA methylation and histone methylation. In addition, *dim*-5 mutant strains are characterized by growth defects and poor fertility. In subsequent complementation experiments evidence was provided that the different reading of a distinct histone methyl mark controls DNA methylation (Collins *et al.*, 2005); introduction of mono- and dimethylation at H3-K9 precluded full complementation of the DNA methylation defect of *dim*-5, but in contrast allowed full complementation of the growth and fertility defects.

A relation between DNA methylation and specific histone lysine methylation was also demonstrated in the fungus *Ascobolus immersus*. However, in contrast to *Neurospora crassa*, dimethylated but not trimethylated histone H3-Lys9 was found to be associated with methylated DNA and changes in the chromatin state were independent of the transcriptional state of the genes (Barra *et al.*, 2005).

The heterochromatin-associated protein 1 (HP1) has been identified as a putative candidate for the recognition of methylation signals. An HP1 homolog is present in *Neurospora* (Freitag *et al.*, 2004) and *Aspergillus* (*hepA*; J. Strauss, pers. commun.). In *N. crassa*, HP1 mutants were devoid of DNA methylation and in addition showed reduced growth phenotypes. The finding that the localization of HP1 is dependent on the catalytic activity of Dim-5 indicated an essential role of HP1 for DNA methylation and led to the hypothesis that the specificity of methyltransferases, in this case Dim-5, might create a mark for the binding/recruitment of proteins (e.g. HP1) to the DNA, which in turn leads to specific methylation of DNA by the direct or indirect recruitment of DNA methyltransferase Dim-2 (Freitag *et al.*, 2004). Moreover, Dim-5 activity itself might be influenced by other modifications such as phosphorylation or acetylation of H3 (Selker *et al.*, 2002).

In *Arabidopsis thaliana*, DNA methylation is also controlled by an H3-K9 HKMT encoded by the KRYPTONITE (SUVH4) gene. KRYPTONITE was identified in a mutant screen for suppressors of gene silencing at the SUPERMAN locus (Jackson *et al.*, 2002). Mutation of the kryptonite alleles reduced cytosine methylation performed by the DNA methyltransferase CMT3. Moreover, CMT3 was demonstrated to interact with an HP-1-like protein (LHP1), which in turn interacts with methylated histones, indicating that DNA methylation might be controlled by H3-K9 methylation, through interaction of CMT3 with methylated chromatin. However, in contrast to *Neurospora*, mutation of LHP1 did not show distinct methylation effects, indicating additional pathways for the mediation of methylation signals (Malagnac *et al.*, 2002).

### Histone arginine methylation: a role in transcription

Methylation of nucleosomal histones by PRMTs plays an important role in diverse processes such as nuclear receptor-mediated transcriptional regulation or chromatin remodeling. In mammals, PRMT1 and CARM1 catalyze asymmetric dimethylation of arginines in histones, which is involved in gene activation, while PRMT5-catalyzed symmetric dimethylation is associated with gene repression (Lee *et al.*, 2005a; Wysocka *et al.*, 2006). HMT1 (also termed RMT1), which is the functional homolog of mammalian PRMT1, has been identified as the major type I PRMT in *Saccharomyces cerevisiae* (Gary et al., 1996). Chromatin immunoprecipitation demonstrated that HMT1 can be cotranscriptionally recruited to highly transcribed genes, indicating that fungal PRMTs such as HMT1 might be involved in gene regulation (Yu *et al.*, 2004). In *Aspergillus nidulans* protein extracts, the PRMT1 homolog RmtA was identified as the predominant *in vitro* activity using histones as substrates (Trojer *et al.*, 2004). Both native and recombinant RmtA proteins possessed H4-R3 specificity and, importantly, methylation of histone H4 by recombinant RmtA affected its acetylation by the HAT p300/CBP, supporting an interrelation of histone arginine methylation and lysine acetylation in transcriptional regulation. Surprisingly, methylation of H3 by RmtB, a PRMT specific for H4-R3 and H3-R26 in *Aspergillus*, had a negative effect on acetylation *in vitro*. It was therefore concluded that methylated H3-R26 might represent a mark for transcriptional repression (Trojer *et al.*, 2004).

However, H4-R3 methylation by PRMT1 homologs is not only restricted to transcriptional activation. Recently, a novel function for arginine methylation in the maintenance of genome stability and the establishment of silent chromatin was reported (Yu *et al.*, 2006). The study demonstrated that *Saccharomyces cerevisiae* HMT1 might be involved in the assembly of the SIR complex, a protein complex important for the formation of silenced chromatin. *HMT1* mutants thereby displayed increased transcription from silent chromatin regions, and increased mitotic recombination within tandem repeats of rRNA gene could be observed. In addition, a decrease in SIR2 and dimethylated H4-R3 distribution across silent chromatin regions was observed.

Formation of symmetric dimethylation of arginines by human PRMT5 is associated with gene repression. For example, PRMT5 methylates H3-R8 and H4-R3 as part of a complex with human SWI/SNF (SWitch/Sucrose NonFermenting) chromatin remodeling components BRG1 (Brahma-Related Gene 1) and BRM (Brahma), which results in the repression of distinct genes (Pal *et al.*, 2004). However, the implication of fungal type II PRMTs in transcriptional regulation remains poorly understood. So far, PRMT5 homologs have been identified in *Aspergillus nidulans* (Trojer *et al.*, 2004) and *Saccharomyces cerevisiae* (Pollack *et al.*, 1999; Lee *et al.*, 2000). HSL7 has a substrate specificity similar to that of PRMT5, indicating functional conservation of the proteins. *In vitro*, methylation of H2A and H4 and bovine myelin basic protein (MBP) was demonstrated for HSL7 (Lee *et al.*, 2000); mono-methylated and/or symmetrically di-methylated arginine was found on histone H2A after treatment with HSL7 (Lee *et al.*, 2000; Miranda *et al.*, 2006). In contrast to the *in vitro* activities reported, no physiological *in vivo* substrate of the HSL7 enzyme has been identified so far (Miranda *et al.* 2006).

Recombinant RmtC of *Aspergillus nidulans* displayed specificity for H4-R3 and H2A (Trojer *et al.*, 2004). On the structural level, RmtC and HSL7 are distinguished from other PRMT5 homologs by a long insert between the last two β-sheets in the β-barrel domain (Komoto *et al.*, 2002; Martin & McMillan, 2002; Krause *et al.*, 2007).

### Possible roles of protein methylation in the regulation of secondary metabolism in *Aspergillus*

Production of SMs by filamentous fungi is of utmost importance with respect to the broad range of useful antibiotic and pharmaceutical activities as well as less desirable toxic activities (Abarca *et al.*, 2004). A putative role of protein methylation in the regulation of secondary metabolism, in particular for sterigmatocystin biosynthesis, a carcinogen biochemically related to aflatoxin and penicillin of *Aspergillus*, has been suggested. Several observations argue for such a role: penicillin production in *Aspergillus nidulans* is differently regulated in PRMT deletion strains compared with wild type (Bauer, Graessle, Brosch; unpublished results). Several genes of the sterigmatocystin gene cluster have been predicted to encode biosynthetic enzymes and regulatory proteins including putative methyltransferases (Minto & Townsend, 1997; Yu & Keller, 2004). A mechanism of gene cluster regulation was uncovered by complementation of an *Aspergillus nidulans* sterigmatocystin mutant that was unable to express *aflR*, a gene in the sterigmatocystin cluster that regulates transcription of the aflatoxin biosynthetic genes. Complementation was acomplished by the gene *laeA* (loss of *aflR* expression), which encodes a nuclear protein with sequence homology to protein methyltransferases (Bok & Keller, 2004). Deletion of *laeA* blocked the expression of metabolic gene clusters, including the sterigmatocystin, penicillin, and lovastatin gene clusters and, conversely, overexpression of LaeA triggered increased penicillin and lovastatin gene transcription. Moreover, a site-specific mutation of the SAM-binding site of *laeA* resulted in a phenotype with similarity to the Δ*laeA* strain, another indication that LaeA may be a protein methyltransferase (Bok *et al.*, 2006). The fact that LaeA regulates multiple clusters may support a coregulation model for clustering, possibly via chromatin remodeling of cluster loci (Yu & Keller, 2004).

## Concluding remarks

Chromatin modifications have emerged as a basic core mechanism in transcriptional regulation. While the enzymes responsible for these modifications have been explored initially with respect to their specificity for nuclear histones, it is now known that nonchromatin and even nonnuclear proteins are targets of these enzymes as well. Therefore, it has to be considered that almost every cellular pathway may be influenced by chromatin-modifying enzymes, and a complex, yet still elusive interdependence between chromatin dynamics and cellular regulation pathways exists. In contrast to the rapidly increasing understanding of epigenetic regulation in higher eukaryotes, little is still known from filamentous fungi. Taking into account that fungi represent an estimated 1.5 million of species, of which the majority are filamentous fungi, a greater research input from fungal organisms in chromatin research is necessary to fully exploit the possibilities of fungi in pharmaceutical production and biotechnology but also to better understand their role in human diseases, as plant pathogens, and as components of plant ecosystems. Data obtained from *Saccharomyces cerevisiae* are often not applicable to filamentous fungi due to the significant differences and the much more complex metabolic repertoire of filamentous fungi.
